# Nature Renders a Verdict

**Published:** 1900-10

**Authors:** 


### NATURE RENDERS A VERDICT.

NATURE has rendered a verdict in a suit for damages in an English Court which effectually settled all quibbling between the medico-legal lights who fought back and forth for many days. A woman brought suit against a cable car company alleging severe injury as a result of a car in which she was a passenger running off the track. Physicians who appeared for her testified they had made careful examinations and found that she had a tumor which apparently was growing. There were six physicians who thus testified. The tumor they said was ovarian or uterine—there was some doubt regarding its exact character. The woman's family physician swore that the tumor had grown to be six times as large since the accident as it was before, and that all the pains and aches, and nervous manifestations were directly attributable to the accident. Things looked bad for the company and it was a very nice clean case from the standpoint of the woman's attorneys, who had placed the damages at a high figure, for the woman had sworn that the tumor was caused by the accident; that it did not amount to anything before. The company's surgeon was then sworn. He, too, had made an examination, and he swore that while the woman had a tumor it probably was an ovarian cyst; that the accident had had no effect upon its growth, and that it would have grown just as rapidly and produced the same results if she had not been on the car which ran off the track. All her troubles, he said were caused by the tumor and not the accident.

The case dragged on and on, but finally came to an end, and both sides retired to await a verdict. While waiting, the woman's pains suddenly increased in violence and she was forced to go to bed. Within a few hours after that she gave birth to a full term child. The judicial result of the trial is not stated.

In reviewing the evidence, however, one is struck by the oddity of the whole proceeding. It is quite probable that the company's surgeon discovered the true cause of the tumor and concealed it to prevent a possible new suit for a different cause, knowing that nature would come to the relief of his company before long.

The cable car has been accused of many crimes, but this is by all odds the most bizarre charge which has been laid at its door.

